# Supplementary material for: Large Scale Process for Low Crystalline MoO3-Carbon Composite Microspheres Prepared by One-Step Spray Pyrolysis for Anodes in Lithium-Ion Batteries
Source: Nanomaterials (Basel). 2019 Apr 3;9(4):539. doi: 10.3390/nano9040539 (PMC6523477; doi:10.3390/nano9040539)
Supplement: Supplementary file 1 [file nanomaterials-09-00539-s001.pdf]

Supporting Information

Large Scale Process for Low Crystalline MoO_3-Carbon Composite Microspheres Prepared by One-Step Spray Pyrolysis for Anodes in Lithium-Ion Batteries

Jung Sang Cho

Department of Engineering Chemistry, Chungbuk National University, Chungbuk 361-763, Korea; jscho@cbnu.ac.kr; Tel.: +82-43-261-2489; Fax: +82-43-262-2380

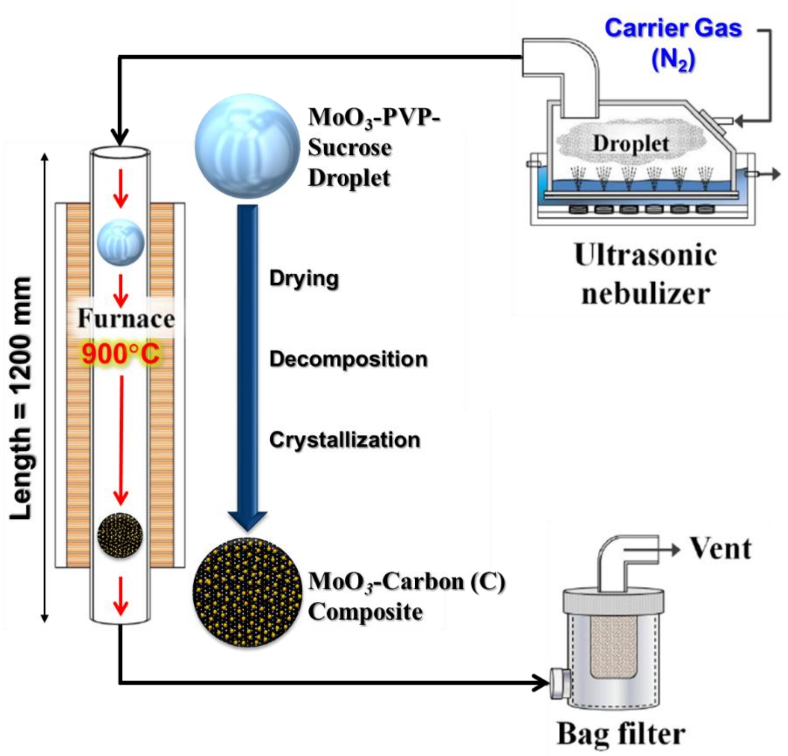

Figure S1. Schematic diagram of spray pyrolysis system applied in the preparation of MoO_3/C composite microspheres.

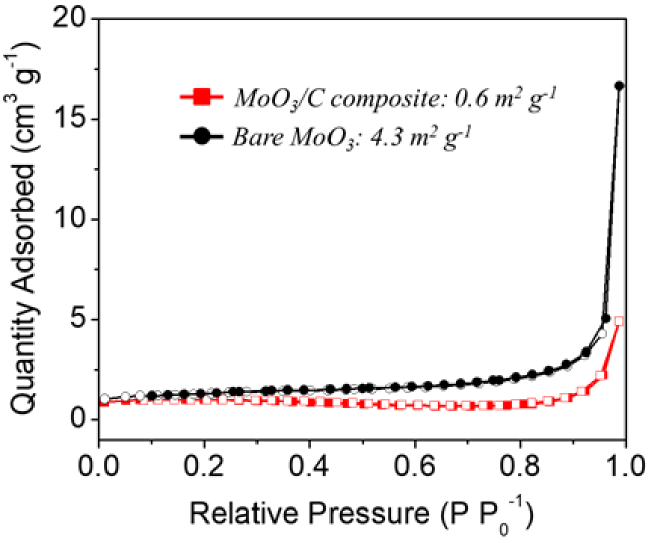

Figure S2. N_2 adsorption-desorption isotherms measured at 77 K for the MoO_3/C composite microspheres and bare MoO_3 powders.

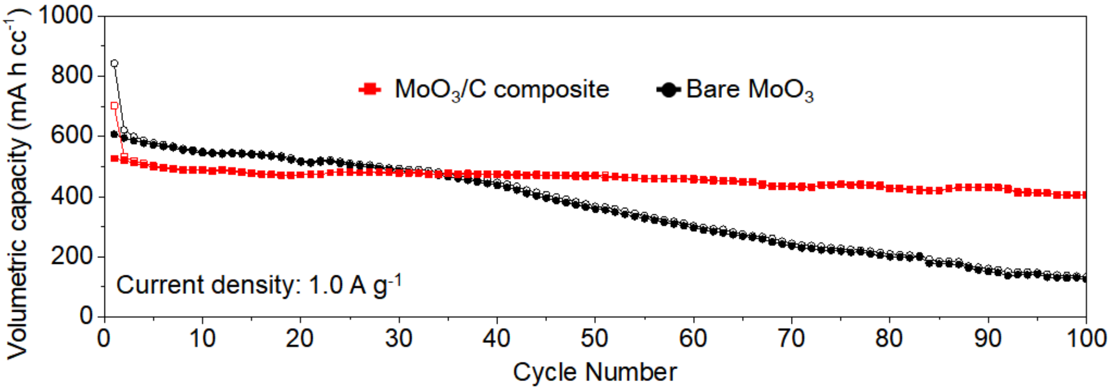

Figure S3. Cycle properties of the MoO_3/C composite microspheres and the bare MoO_3 powders.

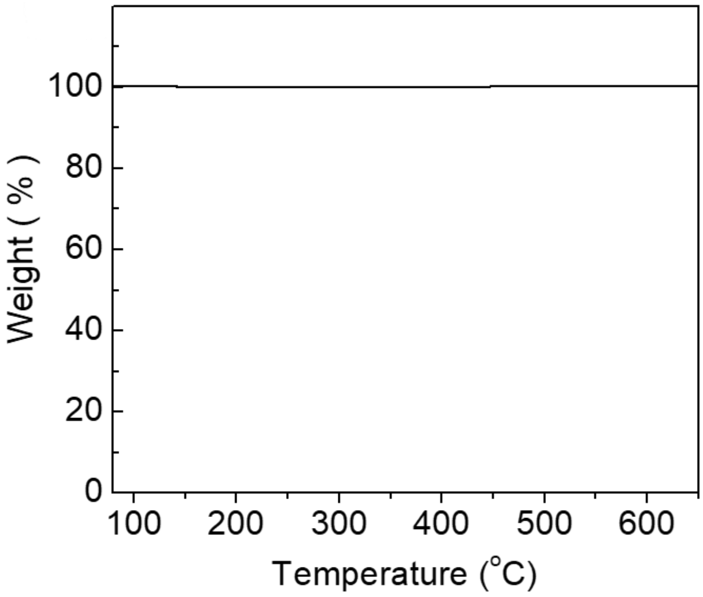

Figure S4. TGA curve of the bare MoO_3 powders.

Table S1. Fitted data obtained from the equivalent circuit for Nyquist plots.

		R_f (Ω)	R_{ct} (Ω)
Fresh cell	MoO_3-C composite	-	26
	Bare MoO_3	-	31
After 1st cycle	MoO_3-C composite	3	18
	Bare MoO_3	5	28
After 100th cycle	MoO_3-C composite	8	42
	Bare MoO_3	82	134
